# HIV/HTLV-1/2 Co-Infection in the Peruvian Amazon: Prevalence and Associated Factors

**DOI:** 10.3390/v18030338

**Published:** 2026-03-10

**Authors:** Wieslawa-Guivanni Alava-Flores, Ivonne Navarro-del-Aguila, Silvia Otero-Rodriguez, José-Manuel Ramos-Rincón, Martin Casapia-Morales

**Affiliations:** 1 Centro de Investigación de Enfermedades Tropicales “Hugo Pesce—Maxime Kuczynski”, Iquitos 16001, Peru; wieslawaalava@gmail.com; 2 Faculty of Pharmacy and Biochemistry, National University of the Peruvian Amazon, Iquitos 16001, Peru; ivonne.navarro@unapiquitos.edu.pe; 3 Infectious Diseases Unit, Doctor Balmis University General Hospital, 03010 Alicante, Spain; o.silvia.r@gmail.com; 4 Institute of Healthcare and Biomedical Research (ISABIAL), 03010 Alicante, Spain; 5 Clinical Medicine Department, Miguel Hernández University of Elche, 03202 Elche, Spain; 6 Faculty of Human Medicine, National University of the Peruvian Amazon, Iquitos 16001, Peru; mcasapia@acsaperu.org

**Keywords:** human T-lymphotropic virus 1, HIV infections, co-infection, prevalence, risk factors

## Abstract

Co-infection with human T-cell lymphotropic virus types 1 and 2 (HTLV-1/2) and HIV is not routinely screened for, yet it may significantly influence clinical progression, mortality, and quality of life in affected individuals. This study aimed to estimate the prevalence of HTLV-1/2 co-infection among adults living with HIV and to identify associated epidemiological factors in the Peruvian Amazon. A cross-sectional study was conducted including patients receiving antiretroviral therapy through the multidisciplinary TARGA program in Iquitos, Peru, during the second quarter of 2013. Screening for HTLV-1/2 antibodies was performed using enzyme-linked immunosorbent assay, with reactive samples confirmed by Line Immunoassay. Demographic and behavioral variables were collected, and prevalence odds ratios with 95% confidence intervals were estimated using logistic regression models. Among the 284 patients included, 28 were co-infected with HIV and HTLV-1/2, resulting in a prevalence of 10% with a 95% confidence interval of 6.5 to 14.1. In multivariable analysis, age over 35 years and having more than 10 lifetime sexual partners were independently associated with co-infection, with prevalence odds ratios of 12.4 and 3.6, respectively. HTLV-1/2 co-infection was highly prevalent among people living with HIV in the Peruvian Amazon, and the main risk factors identified suggest that cumulative exposure and sexual behavior play a significant role in the joint transmission of both retroviruses, supporting the need to consider systematic HTLV screening in endemic settings.

## 1. Introduction

Infection with human T-cell lymphotropic virus types 1 and 2 (HTLV-1/2) represents a major public health challenge in tropical and subtropical regions. These retroviruses, from the *Retroviridae* family, induce immunodeficiency and predispose individuals to various diseases [1,2]. Although most HTLV-1 carriers remain asymptomatic throughout their lives, a significant proportion develop severe and disabling conditions. In addition to adult T-cell leukemia/lymphoma (ATL), HTLV-1 infection is associated with a broad spectrum of inflammatory and immune-mediated diseases, including HTLV-1-associated myelopathy/tropical spastic paraparesis (HAM/TSP), infective dermatitis, uveitis, arthritis, and an increased susceptibility to opportunistic infections due to immune dysregulation [1,2].

Regarding the epidemiology of HTLV, type 1 is predominant in South America, the Caribbean, and Africa, whereas type 2 is mainly limited to indigenous populations and people who inject drugs [3,4]. Both infections share transmission routes with HIV, especially sexual intercourse, transfusions, and vertical transmission through breastfeeding [5].

The coexistence of both retroviruses may alter their clinical progression. Previous studies have shown that HIV/HTLV-1 co-infection is associated with faster progression to AIDS, higher mortality, and shorter survival, even in the presence of high CD4 lymphocyte counts [4,6]. HTLV-1 infection has been associated with immune dysregulation and impaired cellular immune responses, including functional alterations of CD4+ and CD8+ T cells [6], which may contribute to increased susceptibility to opportunistic infections and immune dysfunction.

In Peru, the prevalence of this co-infection is not well understood [7,8], as HTLV diagnosis is not part of routine screening in HIV care programs.

In 2013, the prevalence of HIV in the general population of Peru was estimated at 0.4%, although it was significantly higher in the Amazonian region, with Loreto (Iquitos) reporting some of the highest incidence rates in the country [9,10]. However, it is important to note that higher figures have been reported in the region; for instance, prevalence rates of up to 9.7% have been identified in certain indigenous populations in the Departments of Loreto and Ucayali [11]. Despite these significant findings, the absence of systematic screening for HTLV-1/2 in clinical settings precludes a precise estimation of HIV/HTLV co-infection in the general population.

Thus, this study aims to estimate the prevalence of HIV/HTLV-1/2 co-infection in adults under treatment for HIV at the Loreto Regional Hospital and to analyze the associated epidemiological factors.

## 2. Materials and Methods

### 2.1. Design and Setting

This cross-sectional analytical study took place at the facilities of the STI-HIV/AIDS and Hepatitis B Health Strategy Program at the Felipe Arriola Iglesias Regional Hospital in Loreto, in the city of Iquitos (Peru), from April to June 2013.

### 2.2. Participants

The study population consisted of adults (≥18 years) being treated for a confirmed diagnosis of HIV. At the time of the study, the hospital’s program had a total registry of approximately 600 patients; however, only those who attended their scheduled clinical appointments during the study period (*n* = 306) were assessed for eligibility All eligible patients who attended the clinic during this period and were tested for HTLV were included, constituting a convenience sample. Of these, all patients who met the age criteria and provided informed consent were included. Exclusion criteria were refusal to sign informed consent, minors without authorization, and patients with a terminal prognosis.

### 2.3. Data Collection Procedures, Techniques and Instruments

Each participant completed a structured questionnaire to provide demographic and behavioral data. A venous blood sample (≤5 mL) was collected by venipuncture using the Vacutainer system. Samples were centrifuged at 3000 rpm for 10 min at the hospital laboratory to separate the serum, which was then stored in 2 mL cryovials at −20 °C until processing.

HTLV-1/2 antibodies were screened using a commercial enzyme-linked immunosorbent assay (ELISA) (HTLV-I/II ELISA 4.0; MP Diagnostics, Illkirch, France), which was the assay implemented in the reference laboratory during the study period. According to the manufacturer, this assay has a reported sensitivity and specificity of 100%. All reactive samples were subsequently subjected to confirmatory testing using the INNO-LIA HTLV I/II Score assay (Innogenetics, Ghent, Belgium), following the standard diagnostic algorithm in place at the time. Only samples that were reactive by ELISA and subsequently confirmed positive by INNO-LIA were classified as HTLV-1/2 positive for analytical purposes.

The results were communicated confidentially via email. Due to logistical and budgetary constraints, additional investigations such as screening for *Strongyloides stercoralis* or detection of HTLV proviral DNA by PCR were not performed.

### 2.4. Statistical Analysis

Continuous variables, such as age, CD4 count, and viral load, were dichotomized based on clinical relevance or median values; therefore, normality testing was not required for these categorical analyses. Categorical variables were summarized as frequencies and percentages. The Newcombe method was used to estimate prevalence and 95% confidence intervals (CIs). Variables were compared using the Chi-square test or, when any expected count was less than 5, with Fisher’s exact test.

Odds ratios (OR) were calculated with their 95% CIs for different risk factors, sociodemographic variables, CD4 count, and viral load. A multivariable logistic regression model was fitted using a stepwise forward procedure to identify independent risk factors for HTLV, adjusting for potential confounders such as age, sex, and history of sexually transmitted infections (STIs). Covariates yielding a *p* value of less than 0.10 in univariate analyses were considered eligible for inclusion in the model. Data were analyzed using STATA v12 software.

### 2.5. Ethical Aspects

The Institutional Ethics Committee of the Loreto Regional Hospital approved the study (Report No. 003 CIEI-HRL-2013), classifying it as minimal-risk research. Data confidentiality was strictly maintained, and results were only disclosed to each participant’s HIV healthcare provider, who ensured appropriate follow-up and treatment.

## 3. Results

**Figure 1:**
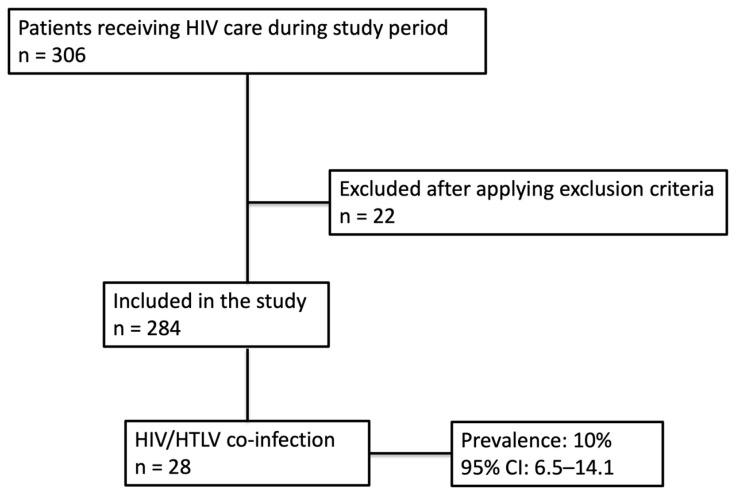
Flow chart of patients.

During the study period, 306 potentially eligible patients were in treatment for HIV at the Felipe Arriola Iglesias Regional Hospital. After applying exclusion criteria, 284 were included, and 28 of these had HIV/HTLV co-infection (prevalence 10%, 95% CI 6.5–14.1) (Figure 1).

### 3.1. General Epidemiological Profile

Most participants were men (71%) and under 35 years of age (55%). Half had their sexual debut before the age of 14 and reported having had more than 10 sexual partners, while 24% had a history of sexually transmitted infections (STIs), and 9% had received blood transfusions (Table 1).

### 3.2. Risk Factors for Co-Infection

In the unadjusted analysis (crude OR) (Table 2), the variables significantly associated with co-infection were age > 35 years, having more than 10 lifetime sexual partners, smoking, history of genital herpes, and low CD4 count (≤172 cells/mm^3^).

**Table 2:** Crude analysis of explanatory variables and their association with HIV/HTLV co-infection.

Variable	Categories	*n*/N (%) [95% CI]	Crude OR (95% CI)	*p*-Value
Age	≤35 years	3/157 (1.9%) [0.6–5.5]	1.00	-
	>35 years	25/127 (19.7%) [13.7–27.5]	12.58 (3.69–42.8)	<0.001
Gender	Female	4/83 (4.8%) [1.9–11.8]	1.00	-
	Male	24/201 (11.9%) [8.2–17.1]	2.6 (0.9–7.9)	0.070
Marital Status	Married/Cohabiting	7/109 (6.4%) [3.1–12.7]	1.00	-
	Single/Divorced/Widowed	21/174 (12.1%) [8.0–17.8]	2.0 (0.8–4.9)	0.130
Community Setting	Urban	21/188 (11.2%) [7.4–16.5]	1.00	-
	Rural	7/95 (7.4%) [3.6–14.4]	0.6 (0.3–1.5)	0.300
Breastfed as a child	No	8/52 (15.4%) [8.0–27.5]	1.00	-
	Yes	20/232 (8.6%) [5.6–13.0]	0.5 (0.2–1.2)	0.130
History of Blood Transfusions	No	3/28 (10.7%) [3.7–27.2]	1.00	-
	Yes	25/256 (9.8%) [6.7–14.0]	0.7 (0.2–2.6)	0.600
Age at sexual debut	≤14 years	14/143 (9.8%) [5.9–15.8]	1.00	-
	>14 years	12/138 (8.7%) [5.1–14.6]	0.9 (0.4–1.9)	0.700
Lifetime sexual partners	0–10 partners	7/143 (4.9%) [2.4–9.8]	1.00	-
	>10 partners	20/140 (14.3%) [9.4–21.0]	3.2 (1.3–7.9)	0.011
History of Blood Transfusions	No	3/28 (10.7%) [3.7–27.2]	1.00	-
	Yes	25/256 (9.8%) [6.7–14.0]	0.7 (0.2–2.6)	0.600
Dental procedure (last month)	No	5/42 (11.9%) [5.2–25.0]	1.00	-
	Yes	23/242 (9.5%) [6.4–13.8]	0.5 (0.2–1.6)	0.300
Acupuncture, tattoos or surgery	No	20/199 (10.1%) [6.6–15.0]	1.00	-
	Yes	8/85 (9.4%) [4.8–17.5]	0.9 (0.4–2.2)	0.800
Ever smoker	No	18/224 (8.0%) [5.1–12.3]	1.00	-
	Yes	10/60 (16.7%) [9.3–27.9]	2.3 (0.9–5.2)	0.050
Drinks alcohol	No	13/148 (8.8%) [5.2–14.5]	1.00	-
	Yes	15/136 (11.0%) [6.8–17.4]	1.3 (0.6–2.8)	0.500
History of STIs (any)	No	18/215 (8.4%) [5.4–12.8]	1.00	-
	Yes	10/69 (14.5%) [8.1–24.7]	1.8 (0.8–4.2)	0.100
Syphilis	No	24/265 (9.1%) [6.2–13.1]	1.00	-
	Yes	4/19 (21.1%) [8.5–43.3]	2.9 (0.9–9.7)	0.080
Gonorrhea	No	25/256 (9.8%) [6.7–14.0]	1.00	-
	Yes	3/28 (10.7%) [3.7–27.2]	1.3 (0.4–4.7)	0.700
Genital herpes	No	22/268 (8.2%) [5.5–12.1]	1.00	-
	Yes	3/9 (33.3%) [12.1–64.6]	5.4 (1.3–23.7)	0.020
CD4 lymphocytes (cells/mm^3^)	≤172	19/141 (13.5%) [8.8–20.1]	1.00	-
	>172	9/140 (6.4%) [3.4–11.8]	0.4 (0.2–1.0)	0.050
Viral load (copies/mm^3^)	≤85,691	11/123 (8.9%) [5.1–15.3]	1.00	-
	>85,691	15/122 (12.3%) [7.6–19.3]	1.4 (0.6–3.2)	0.400

NA: Not applicable, STI: Sexual transmitted infection.

In the multivariate model, two variables maintained an independent association with HIV/HTLV co-infection: age over 35 years of age (adjusted OR 12.4, 95% CI 3.6–43.2; *p* < 0.001) and having had more than 10 sexual partners (adjusted OR 3.6; 95% CI 1.4–9.1; *p* = 0.007).

## 4. Discussion

Our data show a high prevalence of HTLV-1/2 infection (10%) in patients under treatment for HIV in the Peruvian Amazon. This estimate is higher than that reported in the general population in the Iquitos area (around 2.0%) and Peru (approximately 3%) [12,13,14].

On the other hand, our results are consistent with other studies in people living with HIV. In a meta-analysis of three studies in Peru, the prevalence of HIV/HTLV co-infection was 10%, and in other endemic regions of South America, especially Brazil, it ranges from 2% to 23% [7,15,16,17].

In our study, conducted in one of the two hospitals in the city of Iquitos, the prevalence in 2013 was higher than the 4.8% obtained in the recent study conducted in 2023 [8]. The higher prevalence observed in our study may be related to differences in population characteristics, as the 2023 study reports improved disease control and a lower reported number of sexual partners.

The associated factors identified—age over 35 and high number of sexual partners—reflect patterns of prolonged exposure and cumulative risk behaviors. This result coincides with the study conducted in the hospitals of Iquitos, where age was also a risk factor for HTLV acquisition. Previous studies in Brazil and Peru had already pointed to the importance of sexual behavior and history of STIs as major determinants of HTLV transmission [18,19]. In this regard, the high percentage of patients who reported having their sexual debut before the age of 14 and the high number of partners indicate persistent exposure to STIs.

In our study, a history of herpes infection was more frequently observed among co-infected individuals in the unadjusted analysis. However, as no adjusted multivariable logistic regression confirmed this relationship, this finding should be interpreted with caution and cannot be considered an independent association. From a biological perspective, ulcerative infections may facilitate HTLV transmission through mucosal disruption or local inflammation, a mechanism that has been previously hypothesized. Moreover, previous reports have described a higher frequency of skin manifestations and herpes zoster in HTLV-1 carriers [20]. Nevertheless, further studies with appropriate multivariable adjustment are needed to clarify whether herpes infection represents an independent risk factor or merely reflects shared behavioral or immunological vulnerabilities. The immunological impact of co-infection remains a subject of debate [4,6]. While HIV is cytopathic and destroys CD4 lymphocytes, HTLV-1 induces clonal proliferation, which can lead to falsely elevated but functionally ineffective counts [4,6]. In this study, co-infected individuals had lower CD4 cell counts. Although this finding was not fully supported by additional statistical analyses, it may reflect immune dysregulation in the context of HIV/HTLV-1 co-infection, as previously described in the literature [21]. However, given the observational design of the study, no causal inferences can be made regarding the interaction between the two viruses.

It is important to contextualize these findings within the evolution of antiretroviral therapy in Peru. At the time of data collection in 2013, the Peruvian national program primarily utilized first-line regimens based on non-nucleoside reverse transcriptase inhibitors, specifically Efavirenz, combined with nucleoside analogues like zidovudine or lamivudine. Since then, clinical guidelines have transitioned toward integrase strand transfer inhibitors, such as dolutegravir and raltegravir, which offer higher genetic barriers to resistance and faster viral suppression. Consequently, while our study reflects a period where viral load undetectability was lower, current rates of viral suppression in Iquitos are expected to be significantly higher due to these pharmacological advances. These changes highlight the importance of our baseline data to understand how co-infection dynamics might have shifted with more potent modern therapies

One of the main strengths of this study is its focus on epidemiological surveillance strategies for HIV/HTLV co-infection in the Peruvian Amazon. By addressing this neglected area, the study contributes valuable information for public health decision-making and future research planning in the Amazon region.

In contrast, limitations of this study include its cross-sectional design, which prevents establishing causality. Additionally, the study did not systematically collect data on condom use or other sexual risk behaviors, which may have limited our ability to assess the role of preventive practices in HTLV transmission among people with HIV. Furthermore, marital status was not disaggregated into specific categories (e.g., single, married, divorced, widowed), which may represent distinct epidemiological risk profiles and could have provided a more nuanced understanding of transmission dynamics. Similarly, the category “0–10 lifetime sexual partners” was relatively broad and did not allow differentiation of potentially protective factors such as long-term monogamy, thereby limiting a more detailed stratified risk analysis. Moreover, specific neurological manifestations associated with HTLV infection, such as tropical spastic paraparesis/HTLV-1-associated myelopathy (TSP/HAM) or peripheral neuropathy, were not systematically assessed, which precludes evaluation of the clinical burden of HTLV-related neurological disease in this cohort. This precluded a specific sub-analysis of types for the 2013 cohort. However, this gap has been addressed in a subsequent study conducted at the same center ten years later [8], which identified a predominance of HTLV-2 (3.8%) over HTLV-1 (0.3%) among people with HIV.

Moreover, while the sample size was limited to 284 participants, the results provide a crucial baseline for the region. These findings open the door to longitudinal assessments; indeed, the comparison with 2023 data already suggests a shift in the clinical profile, such as the significantly higher age observed in co-infected patients in recent years (55 vs. 39 years), which warrants further investigation into the long-term clinical evolution of HIV/HTLV co-infection.

## 5. Conclusions

The prevalence of HTLV-1/2 co-infection in adults treated for HIV at the Loreto Regional Hospital was high, reaching 10%. in 2013, the year in which the data were collected. Co-infection was significantly associated with age over 35 years and having had more than 10 lifetime sexual partners. These findings underscore the need to implement routine HTLV-1/2 screening in comprehensive care programs for people living with HIV, especially in the tropical regions of Peru. Early knowledge of co-infection status would allow for the optimization of sexual and reproductive prevention strategies adapted to the local context.

## Figures and Tables

**Table 1:** Epidemiological characteristics of patients under treatment for HIV in the Loreto Regional Hospital, April–June 2013 (N = 284).

Epidemiological Characteristics		*n* (%)
Age in years	≤35 years	157 (55.3)
	>35 years	127 (44.7)
Male		201 (70.8)
	Married, cohabiting	109 (38.4)
	Single, divorced, widowed	175 (61.6)
Community setting	Urban	189 (66.5)
	Rural	95 (33.5)
Breastfed as a child		232 (81.7)
Age at sexual debut	≤14 years	143 (50.4)
	>14 years	140 (49.3)
Current sexual partner (yes)		144 (50.7)
N lifetime sexual partners	0–10 partners	143 (50.4)
	>10 partners	140 (49.3)
History of blood transfusion		25 (8.8)
Dental procedure in the last month		25 (8.8)
History of acupuncture, tattoos or surgery		85 (29.9)
Current smoker		60 (21.1)
Drinks alcohol		136 (47.9)
History of sexually transmitted diseases		69 (24.3)
Neurological disorder		4 (1.6)

## Data Availability

The data that support the findings of this study are not publicly available because they contain information that could compromise the privacy of research participants, but are available from the senior author (M.C.-M).

## Abbreviations

The following abbreviations are used in this manuscript:

CI	confidence interval
HTLV	human T-cell lymphotropic virus
OR	odds ratio
STI	sexually transmitted infection
